# The association of the Bolsa Familia Program with children’s oral health in Brazil

**DOI:** 10.1186/s12889-018-6084-3

**Published:** 2018-10-19

**Authors:** Paola Calvasina, Patricia O’Campo, Mateus Mota Pontes, Jamille Barreto Oliveira, Anya P G F Vieira-Meyer

**Affiliations:** 1 Young Talents for Science Program – CAPES, Oswaldo Cruz Foundation (FIOCRUZ-Brazil) Ceará Office, Fortaleza, CE Brazil; 2 Dalla Lana School of Public Health, University of Toronto, Toronto, Canada; 3 Centre for Research on Inner City Health, 209 Victoria Street, 3rd Floor, Toronto, ON M5B1T8 Canada; 4 University of Fortaleza, Av Washington Soares 1321, Edson Queiroz, Fortaleza, CE CEP 60811-905 Brazil; 5 Oswaldo Cruz Foundation (FIOCRUZ- Brazil) Ceará Office, Fortaleza, CE Brazil

**Keywords:** Brazil, Children, Conditional cash transfer, Oral health

## Abstract

**Background:**

Several studies have demonstrated that Conditional Cash Transfer (CCT) programs reduce poverty/inequity and childhood mortality. However, none of these studies investigated the link between CCT programs and children’s oral health. This study examines the association between receiving the Brazilian conditional cash transfer, Bolsa Familia Program (BFP), and the oral health of five-year-old children in the Northeast of Brazil.

**Methods:**

We conducted a cross-sectional study with 230 caregivers/children randomly selected in primary health care clinics in the city of Fortaleza in 2016. Interviews and oral health examinations were performed. Descriptive statistics and multiple logistic regression analyses were conducted to identify factors associated with dental caries among five-year-old children enrolled in the BFP.

**Results:**

Around 40% of children enrolled in the BFP had dental caries. However, those who received Bolsa Familia (BF) for a period up to two years (OR = 0.13, 95% CI 0.05–0.35) had substantially lower adjusted odds of having dental caries than those who had never received BF. In addition, the association of BF and dental caries was more prominent among extremely poor families (OR = 0.05, 95% CI 0.01–0.28).

**Conclusions:**

Although initial enrolment in the BFP predicted low dental caries among five-year-old children, the prevalence of dental caries in this population is still high, thus, public health programs should target BF children’s oral health. An ongoing effort should be made to reduce oral health inequalities among children in Brazil.

## Background

Dental caries is a major public health problem worldwide. It disproportionally affects disadvantaged segments of society, placing an additional health burden on vulnerable groups [1]. Although there has been a decrease in the prevalence of dental caries around the world, this disease still affects more than 621 million children and 2.4 billion adults worldwide [2]. Untreated dental caries can impact individuals’ daily life activities, including eating, sleeping, and social functioning [3–6], and can even compromise employability and social opportunities. Dental problems can also adversely affect society, through work and school absenteeism [6].

In Brazil, large oral health inequalities still exist. For instance, data from the national Brazilian Oral Health Survey (2012) [7] indicates that the decrease in dental decay in five-year-old children was less prominent than in older ages, and with more than 80% of decayed teeth in five-year-old children gone untreated. Results from this National survey also showed large social inequalities in the prevalence of dental caries and access to care among five-year-old children [7].

Several social and public health policies have reduced the effect of income inequalities, which can have an impact on health and access to care in Brazil, with the *Bolsa Família* Program (BFP) and the *Estratégia de Saúde da Família* (ESF; Family Health Strategy) among the most important ones. The BFP is a Conditional Cash Transfer (CCT) program, established in 2003, which seeks to alleviate poverty and reduce inequalities through the provision of cash payments to families (approximately R$85 per person a month) upon compliance with a set of health and education requirements [8]. Health requirements for children younger than seven years old include compliance with immunization schemes, regular health check-ups, and growth monitoring [8]. Regular oral health check-ups are not included in the mandatory health requirement.

The objectives of CCT programs are twofold. First, in the short term, the program aims to alleviate poverty by providing a minimum monthly financial incentive to extremely poor (families with a per capita income of R$85.00 a month—approximately US$22.00) and poor families (families with a per capita income between R$85.00 to R$170.00 a month—approximately US$22.00-US$44.00). Although the program does not completely address poverty issues since it does not ensure the development of skills that will attenuate a family’s low social position, when enforced with other social policies (e.g., education, employment, microcredit), it can be an important resource to enable extremely poor and poor families to escape the vicious poverty cycle [9]. Second, the program encourages health behaviour that will impact overall household well-being, and therefore in the long term contribute to break the poverty cycle [8, 10, 11]. Additionally, CCTs rely on the recognition that there are barriers that prevent individuals from using basic curative and preventive health services, including direct financial costs (when universal care systems are not completely free, or even when they are free, it does not mean universal access), indirect financial costs (e.g., transportation costs), and opportunity costs (time spent accessing health services) [11]. Furthermore, CCTs seek to address cultural barriers such as an individual’s failure to perceive the benefits of complying with CCTs health and education conditionalities [10]. Along these lines, although oral health is not included in the BFP health conditionality, it is possible that the minimum income benefit could reduce the effects of financial strain in the oral health of children by helping families pursue oral hygiene kits (toothbrush and toothpaste) and/or improving the overall food consumption (reducing the cariogenic diet) [12], which are linked to better oral health outcomes. Additionally, caregivers may be better informed and may feel more motivated to take care of their children’s oral health as a result of the BF health conditionality.

In general, the health-related requirements are performed by health team members of the Brazilian ESF in primary health care facilities in the country. The ESF is the first level of primary care in the Brazilian public health care system. The program was introduced in 1994 and has since expanded nationwide. It is organized by Family Health Teams (FHT), which is comprised of family physicians, registered nurses, health assistants, community health workers, family dentists, and nurse and dental assistants. These teams provide comprehensive free-of-charge basic care to registered families in a pre-established territory, including maternal, child care, health promotion, preventive activities, dental care, chronic/infectious disease management, and home visits and referrals [13] . The ESF is one of the largest primary health care systems across the globe, and in 2017 approximately 40,000 family health teams covered 5398 of the 5565 municipalities in Brazil, providing care for roughly 63.2% of the Brazilian population [14].

The evidence about the association of BFP on participants’ health remains equivocal. While some studies have demonstrated that BFP reduced poverty/inequity [15], decreased childhood mortality [16, 17], and improved children’s reported health status [18–21], other studies revealed no difference in the immunization status of children who benefited from this program [22], a small reduction in the rate of weight gain among children from beneficiary households [23], and an increase in consumption of sugar and sugar-sweetened beverages among BFP participants [24] . These data suggest that the positive health effects of CCTs are incremental when they are combined with available and efficient primary health care systems [25, 26].

To date, only three studies have investigated the effect of BFP on children’s oral health [27, 28], with none focusing on the length of enrolment on five-year-old children. Santos et al. (2013) [27] identified a high prevalence of dental caries (78.5%) among four- to eight-year-old children from the Northeast of Brazil (one of the poorest regions of the country) who received BF since birth. Further, Oliveira et al. (2013) [28] in a study conducted in the South of Brazil (one of the richest regions of the country) showed that schoolchildren aged 8 to 12 years old who receive BF had worse oral health outcomes than their counterparts. Petrola et al. (2016) [29], who investigated dental care and oral health promotion activities provided by FHT to children and caregivers enrolled in the BFP, identified that most dentists performed no systematic effort to promote oral health care to BFP children, and that, in general, family health professionals did not develop any oral health activities with these children. Nevertheless, the impact of BFP on children’s dental caries remains largely unknown. In this study, we examine the impact of BFP on five-year-old children’s oral health in a sample of families residing in Fortaleza, Northeast of Brazil.

## Methods

This cross-sectional study was conducted between January and December 2016 in Fortaleza. Fortaleza is the capital of the state of Ceará and the fifth largest city in the country (approximately 2.6 million habitants) [30], situated in the Northeast of Brazil. In 2015, the average monthly income in the city was of approximately 2.7 Minimum Wages [(MW) BRA R$2127.60, approximately US$537], with 30% of residents living below the poverty line (i.e., average per capita income of less than R$140 or US$44) [31].

A sample size of 230 pairs of caregivers/children (alpha = 0.05, power = 80%) was calculated as being necessary to accurately measure caries prevalence, based on reports from the Brazilian National Oral Health Survey on an 86% prevalence of caries in five-year-old children in the city of Fortaleza. Sample size was calculated using the formula for proportion estimation for a finite population size. We used the following parameters: *N* = 20,152; *p* = 0.86; Zα/2 = 1.96 (α = 95%); error = 5%. Therefore, our sample would allow us to estimate prevalence with a 95% confidence level and a 5% error. We used dmft (count of decayed, missing and filled deciduous teeth) to collect data on caries because it is a standard index, recommended by the WHO (2013) [32], and utilized worldwide in oral health surveys. Nonetheless, the results were reported as the prevalence of dental caries, as estimated in the sample size calculation.

The study population included five-year-old children and her/his parent/guardian, whose families are registered in the national unified social system registry (CADUNICO) to receive the BFP benefit. The sample included both children who were registered in the CADUNICO and were receiving BF benefit (BF recipient child) and children registered in the CADUNICO but not yet receiving the BF benefit (BF profile child). Due to confidentiality issues, it was not possible to obtain a roster of participants to serve as a sampling frame. Study participants were, therefore, recruited in 62 randomly selected primary health care facilities in the city of Fortaleza, while attending regular health check-ups. Researchers approached potential participants (caregivers with five-year-old children) and asked them if they were registered in the CADUNICO and had the profile to receive BF or were currently receiving the benefit. Those who answered yes to either of the two questions were asked to participate in the research. Less than 5% of the potential participants did not agree to participate in the research, mainly due to time constraints. Children waiting to receive dental care were not approached to participate in the research due to potential bias on the results, as dental caries was the main outcome of the study. We excluded children with any physical or mental impairments from the sample.

### Measures

Data collection was conducted in two phases. First, trained and calibrated interviewers (three senior undergraduate dental students and one dentist) interviewed enrolled children’s primary caregiver. Interview data included questions on sociodemographic characteristics (e.g., age, gender, household monthly family income, housing situation, water supply), oral health behaviours (e.g., toothbrushing habits), and access to dental care.

Second, upon completion of the questionnaire, children were clinically examined using the dmft and DMFT (count of decayed, missing and filled deciduous and permanent teeth) indices based on WHO diagnostic criteria [32] . Caries experience was measured using a ballpoint probe. Before examination, oral hygiene instructions were provided. Twenty duplicated examinations for inter-examiner agreement with the gold standard (PC, main author) revealed a kappa value of 0.82 for examiner 1, 0.84 for examiner 2, and 0.90 for examiner 3 for primary and permanent dentition, showing a high level of agreement. The intra-examiner kappa value ranged from 0.86 to 0.92.

We estimated income in our model as a measure of per capita income that was calculated using two questions in the survey: household income (categorical variable) and number of residents in the household (continuous variable). We used the method indicated by Celeste and Bastos (2013) [33] to estimate the mean for the upper open-ended category. The income per capita variable was categorized using the BF per income strata (no income, R$85–170, R$170–255, R$255–340, R$340–425).

Other covariates included caregiver’s age (20–25, 26–30, 31–35, 36–40, 41–45, > 45), marital status (married vs. unmarried), level of education (illiterate, elementary school or less, high school or less, university or less), number of people living in the same residency (≤ 2 people, 3 people, 4 people, 5 people, ≥ 6 people), access to fluoride water (yes or no), children’s gender. These covariates were included based on prior literature assessing the association of socioeconomic factors on children’s oral health, and on the impact in the main exposure coefficient [27, 28]. Ethics approval was obtained from the Universidade Estadual do Ceará (Ceará State University).

### Data analysis

Descriptive statistics were calculated. Crude and adjusted analyses between the dependent variable and the independent variables were performed, and odds ratios (ORs) with 95% confidence interval (CI) were estimated. We then estimated ORs using multivariable logistic regression models to predict factors associated with the prevalence of dental caries.

The models were built in the following sequence: Model 1: unadjusted model that examined the association between various independent variables and presence of dental caries, Model 2: adjusted for several independent variables including two socioeconomic position variables (education and per capita income), and access to dental care, Model 3: adjusted for several independent variables including two socioeconomic position variables (education and per capita income) and one variable on reasons for dental visit. Based on literature showing that children’s dental visits were associated with dental caries [34], we decided to explore this association in our study. Thus, a variable reasons for dental visit (never visited the dentist; visited the dentist due to pain, caries, and abscess; visited the dentist for other reasons) was created in order to better explain the relationship between dental utilization and dental caries among children of families who received BF.

The variables included in the models were examined using two approaches: (1) analysing some causal assumptions or beliefs [2, 35] verifying the percentage change (10% limit to consider a variable as a confounder) in the main independent variable estimates from the unadjusted model (bivariate associations) [35, 36].

Based on prior literature showing that a CCT program targeting pregnant women was more effective at improving maternal and neonatal care in the poorest strata of the society [22], we created an interaction term to examine if this relationship would also apply to our main analysis (time of enrolment in the BF and presence of dental caries). Thus, we categorized per capita income in R$0–170; > R$170 that was included in Model 5. We performed all analyses using STATA version 14 (College Station, Texas).

## Results

This study examined the association between receiving BF and five-year-old children’s oral health. More than 70% of the participants received BF (Table 1). Among participants who reported receiving BF, 41% had an income per capita of more than R$170 (data not shown). Most caregivers interviewed were children’s mothers (94.6%), between the age of 31 and 35 years (23%), married (55.2%), with elementary school or less (46.5%), with a household monthly family income of less than one MW (41.7%), and who have been receiving BF for at least two years (30%). Most of the children examined were boys (53.9%). Although over half of caregivers reported that their children needed dental treatment (65.2%), the majority of children had never visited a dentist (63.5%) (Table 1).

**Table 1:** Social, demographic and dental characteristics of families enrolled in a study of BFP and oraL health in Fortaleza, Brazil 2016 (*N* = 230)

Variables	n (%)	Presence of dental caries n(%)	No caries n(%)
Bolsa Familia Recipient (yes)	167 (72.6)	67(40.1)	100(59.8)
		*p* = 0.02	
Years of enrollment in the Bolsa Família Program			
Not recipient	63 (27.4)	36(57.1)	27(42.9)
0–2 years	69 (30.0)	23(33.3)	46(66.7)
2–5 years	57 (24.8)	28(49.1)	29(50.8)
More than 6 years	41 (17.8)	16(39.0)	25(60.9)
		*p* = 0.03	
Mother as main caregiver	175 (94.6)	75(42.8)	100(57.1)
		*p* = 0.30	
Caregivers’ Age			
20–25	35 (15.2)	19(54.3)	16(45.7)
26–30	51 (22.2)	25(49.0)	26(50.9)
31–35	53 (23.0)	24(45.3)	29(54.7)
36–40	36 (15.6)	13(36.1)	23(63.9)
41–45	25 (10.8)	9(36.0)	16(64.0)
46–50	11 (4.8)	4(36.4)	7(63.64)
> 50	19 (8.3)	9(47.4)	10(52.6)
		p* = 0.70	
Marital Status			
Married	127 (55.2)	58(45.7)	69(54.3)
Unmarried	103 (44.8)	45(43.7)	58(56.3)
		*p* = 0.80	
Employment Status			
Unemployed	165 (71.7)	71(43.0)	94(56.9)
Employed	65 (28.3)	32(49.2)	33(50.7)
		*p* = 0.39	
Caregivers’ Level of Education			
Iliterate	9 (3.9)	6(66.7)	3(33.3)
Elementary school or less	107 (46.5)	53(49.5)	54(50.5)
High school or less	106 (46.1)	62(58.5)	44(41.5)
University or less	8 (3.5)	6(75.0)	2(25.0)
		p* = 0.30	
Household Monthly Family Income			
No income	14 (6.1)	8(57.14)	6(42.9)
< 1 Minimum Wage	96 (41.7)	39(40.6)	57(59.4)
1–2 Minimum Wages	95 (41.3)	49(51.5)	46(48.4)
> 2 Minimum Wages	18 (7.8)	5(27.8)	13(72.2)
		p* = 0.2	
Number of Children			
1 Child	70 (30.4)	37(52.9)	33(47.2)
2 Children	69 (30.0)	27(39.1)	42(60.9)
3 Children	56 (24.3)	22(39.3)	34(60.7)
≥ 4 Children	35 (15.2)	17(48.6)	18(51.4)
		p = 0.30	
Five-Year Old Child Attend School (Yes)	224 (97.4)	101(45.1)	123(54.9)
		p* = 0.70	
Never participated in any Bolsa Familia Activities	159 (95.8)	63(39.6)	96(60.4)
		p = 0.30	
Chldren’s gender			
Male	124 (53.9)	58(46.8)	66(53.2)
Female	106 (46.1)	45(42.4)	61(57.5)
		*p* = 0.51	
Children’s oral health and acess to			
Self-reported dental needs (yes)	150 (65.2)	80(53.3)	70(46.7)
		*p* < 0.01	
Children has never visited a dentist	146 (63.5)	58(39.7)	88(60.3)
		*p* = 0.04	
Visited a dentist less than six months	47 (56.6)	27(57.4)	20(42.5)
		*p* = 0.50	

*Fisher's-exact-test

The results of the multivariate analysis examining factors associated with dental caries in five-year-old children registered in the CADUNICO are shown in Table 2. In comparison to those who have never received BF, the OR for having dental caries for those who have been receiving the benefit for at least two years was 0.13 (95% CI 0.05, 0.35). Also, compared to the youngest caregivers (ages 20–25) adjusted OR for children from caregivers aged between 41 and 45 years old was 0.17. Children raised in families with a household monthly family income higher than 2 MW had lower adjusted odds of having dental caries than children raised in families with no income (Model 3, Table 2).

**Table 2:** Predictors dental caries among five-year-old children registered in CADUNICO to receive BF

	Model 1 OR (unadjusted, 95% CI)	Model 2* OR (adjusted, 95% CI)	Model 3* OR (adjusted, 95% CI)	Model 4 OR (adjusted, 95%CI
Years of BF enrollment				
Not recipient	1.00	1.00	1.00	1.00
0–2 years	**0.37(0.18–0.76)**	**0.15(0.06–0.39)**	**0.13(0.05–0.35)**	0.94(0.30–2.88)
2–5 years	0.72(0.35–1.49)	0.49(0.20–1.22)	0.53(0.21–1.34)	1.92(0.63–5.87)
More than 6 years	0.48(0.21–1.07)	**0.27(0.10–0.78)**	**0.29(0.10–0.82)**	2.14(0.62–7.38)
Caregivers’ Age				
20–25	1.00	1.00	1.00	1.00
26–30	0.81(0.34–1.92)	0.51(0.17–1.50)	0.59(0.19–1.80)	0.95(0.35–2.55)
31–35	0.70(0.29–1.64)	0.42(0.14–1.22)	0.44(0.14–1.32)	0.62(0.23–1.67)
36–40	0.48(0.18–1.23)	0.31(0.09–1.00)	**0.29(0.08–0.99)**	0.44(0.15–1.35)
41–45	0.47(0.16–1.36)	**0.16(0.04–0.60)**	**0.17(0.04–0.67)**	0.42(0.12–1.44)
46–50	0.48(0.12–1.94)	**0.15(0.23–0.92)**	0.17(0.29–1.01)	0.59(0.12–2.85)
> 50	0.76(0.25–2.32)	0.31(0.06–1.53)	0.31(0.06–1.60)	0.58(0.15–2.29)
Per Capita Income				
No income	1.00	1.00	1.00	
R$ 85–170	**0.41(0.19–0.89)**	**0.25(0.10–0.65)**	**0.28(0.10–0.75)**	
R$ 170–255	1.92(0.74–4.97)	1.20(0.66–6.06)	1.20(0.63–6.33)	
R$ 255–340	1.19(0.48–2.94)	1.15(0.40–3.32)	1.33(0.44–4.01)	
R$ 340–425	0.58(0.23–1.49)	**0.30(0.09–0.94)**	0.31(0.09–1.05)	
> R$ 425	**0.17(0.03–0.89)**	**0.05(0.01–0.33)**	**0.06(0.01–0.44)**	
Caregiver’s Level of Education				
Illiterate	1.00	1.00	1.00	
Elementary school or less	2.04(0.48–8.57)	1.76(0.27–11.33)	1.32(0.20–8.62)	
High school or less	1.41(0.34–5.98)	0.68(0.10–4.72)	0.49(0.07–3.52)	
University or less	0.67(0.08–5.54)	0.12(0.08–1.93)	0.08(0.04–1.72)	
Children’s visited dentist				
No	1.00	1.00		
Yes	**1.75(1.01–3.01)**	**2.71(1.28–5.77)**		
Reason for dental visit				
Never visited the dentist	1.00		1.00	1.00
Visited the dentist due to toothache, caries or dental abscess	**6.33 (2.26–17.77)**		**15.77(3.73–66.63)**	**12.20(3.16–47.01)**
Visited the dentist for other reasons	1.35 (0.71–2.57)		1.69(0.74–3.85)	1.33(0.67–2.64)
Percapita Income				
> 170(R$)	1.00			1.00
0–170 (R$)	1.12(0.72–1.74)			**4.31(1.26–14.77)**
Interaction terms				
Years of BF enrollment x Per Capita Income				
Not BF recipient with > 170(R$)				1.00
0–2 years BF recipient with 0–170(R$)				**0.05(0.01–0.28)**
2–5 years BF recipient with 0–170				**0.11(0.02–0.59)**
More than 6 years with 0–170(R$)				**0.06(0.09–0.37)**
Pseudo R2		0.20	0.24	0.16
AIC		300.3	289.2	289

*Adjusted OR to access to fluoride water, frequency of teeth brushing, children’ sex, oral hygiene instruction, marital status

Statistical significant (*p*<0.05) are in bold

The adjusted OR for having dental caries among those children who reported visiting a dentist was 2.71 (95% CI 1.28, 5.77). We investigated reasons for the association between visiting a dentist and presence of dental caries (Model 2, Table 2). Thus, another model (Model 3, Table 2), was conducted, in which we created a variable including reasons for dental visits (never visited the dentist; visited the dentist due to pain, caries, and abscess; visited the dentist for other reasons). In this model, we found that the adjusted OR for having dental caries among BF children who reported visiting a dentist due to toothache, caries, or dental abscess was 15.77 (95% CI 3.39, 48.11).

The association between BF and dental caries by income strata was also tested (Model 3, Table 2). In this model, the OR for children from poor families (per capita income R$0–170) compared to families whose individuals have a per capita income of more than R$170 was 4.31 (1.26–14.77). However, looking at the interaction term, we found that children from poor families (per capita income R$0–170) who received BF for up to two years were less likely to have dental caries than children from families with a per capita income of more than R$170 monthly not receiving the benefit [OR = 0.05; 95% CI 0.01–0.28]. We estimated the total interaction effect, obtaining the following results for per capita income lower than R$170 [(no BF OR = 4.30; BF < 2 years OR = 0.21; BF 2–5 years OR = 0.47; BF > 6 years OR = 0.25)].

## Discussion

This is the first study examining the oral health of five-year-old children registered in the CAUNICO. We found that 41% of participants with a per capita income greater than R$ 170, reported receiving BF. Based on the program guidelines, this circumstance should not occur. We speculate that many poor families may have income that is not officially declared (e.g. temporary work) and because of that, they are kept in the program. These inconsistencies were also found in previous studies examining coverage and focus of BFP [37, 38].

Our analysis suggests that children whose families were early recipients of BF, receiving benefits for up to 2 years, had 78% lower odds of developing dental caries than non-recipients. This result indicates that BF, in its initial years, acted to prevent dental caries among five-year-old children. Several studies have indicated that CCTs positively impact children’s health, including birth weight, illness, and morbidity [11, 16–21] . Yet, the mechanisms by which CCT programs benefit the health of the population remain unclear. Some authors have argued that the benefit only occurs when CCT programs are combined with a coordinated primary health care system [12, 17]. Our results contribute to the current debate on this issue.

Although free-of-charge dental care is available in the Brazilian primary health care system, through the country’s FHP [13, 14], regular oral health check-ups are not part of the BF health conditionality. Thus, there are no incentives for children registered in the BF to visit a dentist. In fact, our results showed that more than 63% (*n* = 146) of our sample had never visited a dentist, even though the majority of participants reported regularly visiting the health unit (due to BF health conditionalities). Further, a recent study conducted with the primary health care teams in Fortaleza demonstrated that most dentists performed no systematic effort to promote oral health or dental care to children and their caregivers covered by the BFP because they felt that it was beyond their responsibilities [29] . Two possible explanations may help interpret our findings. Firstly, it can be postulated that by having access to health care services, including regular clinical appointments with nurses [29], as part of the BF health conditionality, caregivers may be better informed and feel more motivated to take care of their children’s oral health. Secondly, BFP is likely to improve children’s oral health by enhancing the quality of daily food intake. These explanations are supported by the most recent systematic review examining the effect of the BFP on nutritional outcomes [12], in which a positive association between this program and increased consumption of different food groups and food and nutritional security was found.

Another possible explanation for low caries among children registered in the BFP is that a small increase of income may reduce the impact of stress-related factors on children’s oral health. Living in poverty is associated with a high degree of stress. Studies showed that children in poverty are more likely to experience an unsafe and unhealthy home and to reside in families characterized by psychosocial risk factors such as high levels of conflict, violence, and family turmoil, and low levels of cohesiveness and warmth, [39, 40]. As a result, children growing up in low-income homes are understood to experience higher levels of stress than their counterparts [39, 40]. The pathways explaining these relationships are not completely understood. Nevertheless, one of the possibilities recently contemplated in the literature is related to hormone secretion [41, 42]. Children growing up in poverty may show early signs of allostatic load, including elevated secretion of cortisol and epinephrine, and higher blood pressure [40].

With regards to oral health, Boyce et al. (2006) [43] examining stress-related psychobiological processes that might account for disproportionate rates of dental caries among kindergarten children (five to six years old) in the US identified that a low family social position was associated with financial stress, basal activation of the child’s hypothalamic-pituitary-adrenocortical axis, and higher counts of oral cariogenic bacteria [43]. These authors also showed that: 1) cariogenic bacteria and salivary cortisol secretion were both independently associated with the presence of caries; 2) the highest risk of dental caries was among children with high levels of both salivary cortisol and cariogenic bacteria; and 3) cortisol reactivity to stress was associated with thinner, softer, and thus more vulnerable enamel surfaces. Additionally, maternal allostatic load as a measure of exposure to chronic stress—has been linked to adverse caretaking behaviours, such as taking a child to the dentist, and correlated with presence of caries in children [44]. Thus, the convergence of psychosocial, infectious, and stress-related biological processes appears to be implicated in the production of greater cariogenic bacterial growth, increased physical vulnerability of the developing dentition, and poor oral health care behaviours, and hence dental caries.

The association between receiving BF and oral health was even more prominent among extremely poor families. The ORs from children of families with a per capita income lower than R$170 were 95% lower than those not receiving BF with a per capita income greater than R$170. This finding suggests that BF is more effective in the poorer strata of the population. Similarly, Amudahan et al. (2013) [26] found that the India CCT (Janani Suraksha Yojana) had a higher impact on institutional birth deliveries among a poorer section of intended CCT beneficiaries. Thus, CCT programs appear to mitigate the impact of inequalities on children’s well-being.

In the present study, a large proportion of five-year-old children registered in the BFP had never visited a dentist (63.48%), yet 44.11% of the sampled children had untreated dental caries. Children who reported visiting a dentist had higher odds of having dental caries than their counterparts, an aspect that was explained by analysis showing that our sampled children would mainly visit the dentist when in pain. This suggests that these children are also not receiving proper preventive nor restorative dental care, as the proportion of dental caries is still high among the sampled children. These results are similar to Petrola et al.’s (2016) [29] research conducted with a similar sample of participants in the city of Fortaleza, in which it was shown that more than 60% of children 7 years old or less, enrolled in the BFP, had never received fluoride therapy or participated in oral health education activities and almost 78% had never visited a primary health care dentist. Petrola et al. (2016) [29] also found that one third of the family health dentists interviewed did not provide dental care to children covered by the BFP, as they reported that this care was beyond their responsibilities. Our findings are somewhat worrisome. Childhood caries affects a child’s general health, learning ability, quality of life, and can have a life-long impact on oral health [45, 46] . Children who experience caries as infants or toddlers are at greater odds of developing further caries in their primary and secondary dentitions [45] .

Comprehensive public policy programs can help prevent children’s dental caries among low-income families. For instance, the Early Head Start program in the US, which targeted low-income families and has an oral health component, including daily teeth brushing activities and dental check-ups, has improved teeth brushing among enrolled children [47], improved access to dental care [48], and increased preventive/diagnostic/restorative dental care utilization [49] . Therefore, we speculate that the inclusion of oral health in the BF health conditionality will likely improve children’s oral health outcomes. The oral health teams, which are integrated in the ESF, are one of the largest primary dental care models across the globe. They operate in 4937 of the 5570 Brazilian municipalities, caring for approximately 72 million individuals (roughly 35% of the population) in 2017 [14]. Such extensive dental care network could provide systematic comprehensive dental care for children enrolled in the BFP, especially if oral health were included in the BF health conditionality.

While some authors have argued against the BFP (because the mandatory conditionalities could potentially breach unconditional rights to citizenship), thus supporting Unconditional Cash Transfer (UCT) programs [50], several studies have shown important effects of the BFP on the health outcomes of Brazilian children [11, 12, 17–21]. Alves and Escorel (2013) [51] examined the social impact of BFP among low-income families and identified that the program contributed to families' social inclusion, especially in the economic and social dimensions. Nevertheless, it is known that BFP has limitations regarding work or political civil engagement of participants [50]. Both programs (UCT and CCT) have advantages and limitations. Yet, it is unclear which one would be the best option for the Brazilian context. Thus, further research is needed to compare social, economic, and health outcomes of UCT and CCT programs in Brazilian context.

This study is not without limitations. First, our findings are based on a cross-sectional analysis, which prevented us to establish a causal pathway. However, our analysis provides a first insight into the association of BFP on five-year-old children’s oral health. This analysis is part of a larger research aiming to analyse this relationship longitudinally. Second, our analysis grouped individual recipients and non-recipients of BF. This grouping may underestimate the heterogeneity within each group. However, we were able to classify, in our final analysis, participants by length of enrolment in the BFP, a strategy that may remedy this limitation. Third, participants were included based on their self-reported enrolment in the BFP. However, it is known that 73% of residents in the northeastern region of Brazil with a monthly household income of less than 1 MW (R$788.00) are assisted by FHT in public primary health care units in the region [52]. Thus, it was likely that we would have found our target population among patients assisted by FHT.

## Conclusions

The results of our study provide evidence that the Brazilian CCT health program, despite not having oral health care requirements, is associated with less dental caries among those enrolled in the first 2 years of the BFP as well as after six. These results suggest that the increased income and overall health conditionality are associated with improvements in children’s oral health. Additionally, the data show a high prevalence of dental caries among poor five-year-old children (enrolled in CADUNICO), demonstrating the need of public health policies targeting this specific population.

## Abbreviations

BFP: Bolsa Família Program
CADUNICO: Cadastro Unico
CCT: Conditional Cash Transfer
DMFT: Decayed Missed Filled Teeth
ESF: Estrategia Saúde da Familia
FHT: Family Health Team
MW: Minimum Wage
WHO: World Health Organization
